# Infant Parent Support (IPS): a multidisciplinary intervention to improve the mental health of children with a social worker - a study protocol for a feasibility randomised controlled trial with embedded process evaluation

**DOI:** 10.1186/s40814-025-01616-6

**Published:** 2025-06-05

**Authors:** Jaycee Pownall, Karen Crawford, Lindsay Dalgarno, Judith Fisher, Sharon Graham, Fiona Turner, Helen Minnis, Kathleen Boyd, Abril Seyahian, Alex McConnachie, Nicola Cosgrave, Matt Forde, Carol Atkinson, Janet McCullough, Kapil Sayal, Dennis Ougrin

**Affiliations:** 1 School of Health and Wellbeing, University of Glasgow, Glasgow, UK; 2 South London and Maudsley NHS Foundation Trust and Julia Donaldson, NSPCC, Glasgow, UK; 3 NSPCC, London, UK; 4 East London Family Court, London, UK; 5 Glasgow City Health and Social Care Partnership, Children’S Services, Glasgow, UK; 6 Unit of Mental Health & Clinical Neurosciences, Institute of Mental Health, School of Medicine, Nottingham, UK; 7 Youth Resilience Unit, WHO Collaborating Centre for Mental Health Services Development Centre for Psychiatry and Mental Health, Wolfson Institute of Population Health, Mary University of London, London, Queen UK

**Keywords:** Children in need, Co-production, Feasibility randomised controlled trial (fRCT), Health economics, Infant mental health, Lived experience, Resilience, Social care, Vulnerable families

## Abstract

**Background:**

In many families where children have a social worker, parents have experienced challenges in their own childhoods or have neurodevelopmental conditions. These families often endure significant stress, which is frequently worsened by financial or housing challenges. This added pressure can strain relationships and increase the risk of child maltreatment, as well as contribute to mental health issues in children. Relationship-focused interventions show promise in preventing child maltreatment, although there are currently no interventions that simultaneously address neurodevelopmental conditions and the impact of poverty. We have co-produced, alongside parent experts-by-experience, local stakeholders, and infant mental health practitioners, a new service called Infant Parent Support (IPS). IPS will i) adopt a relationship-focused approach to comprehensive understanding of family functioning, ii) incorporate child and parent mental health and neurodevelopmental awareness, and iii) ensure a poverty aware approach throughout. The aim of this phase is to investigate the feasibility of a definitive Randomised Controlled Trial (RCT) of IPS compared with services-as-usual (SAU).

**Methods:**

The study settings are social care services in two local authorities: Glasgow City Council (Scotland) and the London Borough of Bromley (England). Our target population is children on a ‘child in need’ plan (or the Scottish equivalent) and eligible participants are families where i) the infant(s) are aged 0–5 years and ii) the family has an allocated social worker plus a multi-agency support plan. Thirty participants will be identified by social workers and randomised to receive either IPS or SAU. Families randomised to IPS will receive an intensive multidisciplinary attachment-focused assessment that provides a foundation for relationship-focused interventions. IPS will incorporate child and parent mental health and neurodevelopmental awareness and ensure a poverty aware approach throughout. Families randomised to SAU will receive the assessment and support that social care services normally implement. We will utilise a pre-post and 3/6-month follow-up design with embedded mixed-method process evaluation and exploratory economic analysis. The primary objective is to assess if enough families can be recruited, randomised, and retained in the trial such that a full-scale RCT is likely to be feasible. The secondary objectives are to assess the acceptability and feasibility of the planned outcome measures and the IPS intervention to families and professionals.

**Conclusions:**

A service like IPS, that uses a relationship-focused approach to child and parent mental health, neurodevelopmental and money/housing problems, has never previously been tested. Therefore, there are several areas of uncertainty that need to be addressed before moving onto a definitive RCT.

**Trial registration {2a and 2b}:**

Registered in ClinicalTrials.gov Identifier: NCT06003582. Co-production and Feasibility RCT of Intervention to Improve the Mental Health of Children with a Social Worker. Registered 22/08/2023. https://classic.clinicaltrials.gov/ct2/show/NCT06003582.

## Administrative Information

Note: the numbers in curly brackets in this protocol refer to SPIRIT checklist item numbers [1]. This has been supplemented with the items from the CONSORT extension for randomised feasibility trials, as recommended by Thabane and Lancaster [2].

## Background and Rationale {6a}

It is estimated that in the United Kingdom (UK) 1.6 million children needed a social worker between 2012 and 2018, equivalent to 1 in 10 children [3]. Reasons for social care use are multifaceted and often interlinked, encompassing both child and family level variables, as well as system-level or societal determinants. Social work may become involved with a family when concerns about a child’s safety and well-being are raised, which may be a result of domestic abuse, alcohol and drug misuse, and parental and/or child mental health, physical disability, learning disability, and/or neurodevelopmental conditions (NDC; e.g. attention-deficit/hyperactivity disorder [ADHD] or Autism). The presence of any one of these difficulties, however, will not necessarily have an adverse impact on the welfare of a child, nor can any of these factors be understood without considering the social and economic context in which these issues are experienced [4].

Parents whose children have a social worker often have their own histories of adversity, such as childhood abuse, neglect [5, 6]. Socioeconomic challenges, including low income and parental unemployment, further amplify stress and are linked to poorer mental health outcomes in children [7]. These factors, compounded by the stigma of poverty, strain family relationships and can increase the risk of child maltreatment [8]. In turn, this can exacerbate children's mental health and behavioural issues [9], creating additional burdens for families, services, and society [8, 10]. The impact of childhood adversity extends far beyond immediate hardship, leaving lasting effects on psychological, social, and behavioural development, even when family involvement with social services is brief [3]. Without intervention, a vicious cycle can occur that risks the child being taken into care, a pattern that can persist across generations [11]. However, recent research suggests that providing support to families much earlier could help break this cycle [12]. Short, relationship-focused interventions can greatly improve parental sensitivity if offered soon enough in a child’s life [13], which can significantly reduce the long-term costs to society [14].

Despite there being several randomised controlled trials (RCTs) and protocols of ongoing trials exploring the effectiveness of interventions aiming at preventing child maltreatment ([15–22]; see also Euser et al. [23]) the evidence supporting sustained differences between the intervention and trial arms is insufficient. Parental needs, such as mental health, are rarely addressed [17] and many of these studies have been conducted in areas such as the US, with very different social care contexts compared to the UK [24]. There are suggestions of improvements in parenting behaviours [25] and rates of maltreatment when relationship-focused interventions were used, especially if involving child protection services [20].

We therefore propose to test a newly developed service called Infant Parent Support (IPS) that has been co-produced with parents who themselves have had children involved with social work (Pownall, Dalgarno, Graham, Crawford, Minnis: Lessons learned from co-producing a new intervention: the perceptions and experiences of professionals involved in the co-production of a support service to improve the mental health of children aged 0–5 with a social worker, in preparation). IPS will address three important components that have not previously been tested within one intervention: firstly, IPS will adopt a relationship-focussed approach to comprehensively understand family functioning, secondly, it will incorporate child and parent mental health and neurodevelopmental awareness and thirdly, ensure a poverty aware approach throughout.

### A Relationship-Focused Approach

The IPS intervention will build on the relationship-focused infant mental health approach already used in our ongoing NIHR-funded Best Services Trial (BeST^?^) [26]. In BeST^?^, families randomised to Infant and Family Team (IFTs), based on the New Orleans Intervention Model (NIM) [27], receive an intensive multidisciplinary attachment-focused assessment that provides a foundation for relationship-focused interventions where possible [28]. By incorporating a focus on parents' needs, the intervention also addresses how their own childhood experiences may hinder responsive parenting and empowers them to make changes that could improve their child’s mental health [29]. BeST^?^ is testing whether the IFT approach is effective in a UK context, and follows promising research findings from New Orleans, US, where the model on which IFTs are based originated [27, 29]. Although IFTs infant mental health approach is unique in the UK [30] until now it has focused on children removed from care of biological parents (i.e. foster/kinship care). In addition, it does not explicitly incorporate poverty aware practice. We hypothesise that using such an approach earlier in the family’s development (i.e. before child protection proceedings) will have an enormous potential for change. We hypothesise that intervening to support parents in building family resilience before a crisis precipitates a child coming into care – with an emphasis on understanding, respect, reducing stressors and improving resilience – may give parents a much greater opportunity for change [31]. The importance of identifying and supporting families when problems or risks first emerge aligns with current social care [32, 33] and judicial policy [34] nationally. For instance, the English Care Review Case for Change states that “*too often we are allowing situations to escalate and then being forced to intervene too late, severing children’s relationships and setting them on a worse trajectory*” (p. 10) [11]. The Scottish Care Review concluded that “*where children are safe in their families and feel loved they must stay – and families must be given the support together to nurture that love can overcome the difficulties which get in the way*” (p. 9) [33].

### Mental Health and Neurodevelopmental Awareness

People who have experienced maltreatment are at increased risk of having neurodevelopmental conditions (e.g., ADHD, intellectual disability [ID], and autism spectrum disorder [ASD]). Maltreatment does not appear to cause these conditions [9]: NDCs are highly heritable [35, 36]. However, symptoms of children’s NDCs (e.g., hyperactivity, impulsiveness in ADHD, insistence on routines, sensory sensitivities in ASD) and associated emotional dysregulation can make parenting very challenging – more so if the parent has similar difficulties. If stressed, family relationships can deteriorate, and both parents and children are more likely to experience a decline in their mental health [37–39].

There are many possible factors that can delay the diagnosis of NDCs, including presenting problems being viewed as related to attachment difficulties, trauma associated with social issues [40] or life experiences, as well as resources impacting on access to specialist ND diagnostic services. Nonetheless, identifying NDCs is crucial to improve long term outcomes for children [41, 42]. In addition, without adequate support for parents of children with a NDC, parental stress may escalate [43] increasing the likelihood of the parent developing persistent mental health challenges and/or substance misuse [4]. Unfortunately, availability of services and resources means that often when families have asked for help, child protection investigations have taken priority without the opportunity for intervention or support [44] and judgements about parenting capacity are usually not based on validated assessments [45]. Neurodivergent families may also face accusations that they are inducing, embellishing, or fabricating the illness/condition that they are asking for support for [46]. Current interventions aimed at preventing child maltreatment do not focus on child/parent NDC and frequently fail to consider the impact of parental mental health (for an exception see Mattheß et al. [17]).

### Poverty Awareness

Poverty impacts individuals both directly, through material deprivation and systemic inequalities [47], and indirectly by contributing to parental stress, health challenges, and exposure to poor living conditions [48]. Assessments in child protection aim to provide a holistic understanding of a child's situation, yet they do not always sufficiently address poverty [49, 50], for instance challenges in material circumstances [51] and inadequate housing [52], with little therapeutic support being offered to improve family wellbeing [53]. A poverty-aware approach recognizes the challenges and insecurities families experience, including systemic issues like classism and racism, and their effects. It works alongside families to enhance their situations, empowering them to nurture and care for their children to the fullest [54].

A family’s economic circumstances can also impact on their engagement with supports and services, for instance through the stigma of living in poverty and the direct impact of low income itself [55]. Professionals' personal values and beliefs about poverty can contribute to stigma and exclusion, intensifying the shame experienced by families [56]. This shame can diminish trust in others and hinder the development of close relationships [57]. A poverty-aware approach, by encouraging professionals to engage with families with empathy and respect [58, 59], can help counteract these feelings of shame and create a strong foundation for successful, relationship-based interventions [54]. In addition, when parents or caregivers cannot meet their family’s basic needs, such as providing food for their families, heating their home, and paying bills, emotional distress increases [59] and it becomes harder to attend to or address less imminent issues [60]. For interventions to be accessible, they must address a family’s basic needs such as hunger, childcare, and transport [55]. This could include connecting families to support services like foodbanks and financial assistance [55], or offering families food and drink prior to engaging with services /interventions. Unfortunately, interventions aimed at preventing child maltreatment do not give sufficient attention to the impact of poverty on outcomes [55].

We therefore propose a new co-produced approach that will address these three crucial elements. We will i) use an infant mental health approach to offer struggling families a thorough relationship-focused assessment, ii) identify any NDCs and/or mental health problems that parents and child(ren) might have, and iii) identify any material challenges families are facing – and use this information to respectfully offer timely, poverty-aware, relationship-focused interventions.

## Foundational Work

This article describes the protocol for a feasibility RCT of IPS, an intervention to improve the mental health of children with a social worker. This is the second phase within the Partnership for Change project [61], of which a substantial amount of foundation work has already been completed. Intensive mapping of the current services landscape for struggling families in Glasgow and Bromley [62] and the co-production of IPS is already complete. The current paper focuses on the Feasibility RCT of IPS versus local authority (LA) facilitated services-as-usual.

### Mapping and Modelling of Services

Early in the intervention development, in-depth interviews and focus groups were carried out with numerous stakeholders, including parents with lived experience of child and family social work, health and social care workers, and key policy leaders [62]. The focus of this work was to gain a greater understanding of infant and parent journeys through services when social work services become involved and mapping the statutory and voluntary sector services to optimise the intervention’s usefulness for families, and creating new multi-agency partnerships.

### Intervention Co-production

Co-production is an approach that aligns well with early interventions aimed at improving outcomes for children or preventing escalating need [63]. Although there is wide variation in how co-production has been defined and applied in the literature [64], the current study draws upon the definition provided by the Social Care Institute for Excellence (SCIE), stating it is “*a way of working whereby citizens and decision makers, or people who use services, family carers and service providers work together to create a decision or service which works for them all. The approach is value-driven and built on the principle that those who use a service are best placed to help design it*” (p. 6 [65]).

The involvement of people using services in designing and delivering support has far reaching benefits to individuals, professionals, services, as well as wider practice and policy [63–70]. Although at the heart of social care is benevolence, risk avoidance and risk regulation often take precedence [71, 72]. This can lead to unequal power in relationships, thus preventing meaningful engagement and access to care. Parents involved with social services frequently report feeling powerless when accessing services, and fear that asking for help will result in their child being removed [73, 74]. Co-production can inform children’s social care work, offering an opportunity for seldom heard groups to have their voices elevated and to shift the power balance between people who use the service and service providers [75]. It can help to reduce stigma, not only when accessing services and support, but also challenging stigma and discrimination that may arise from professionals [63].

**Fig. 1:**
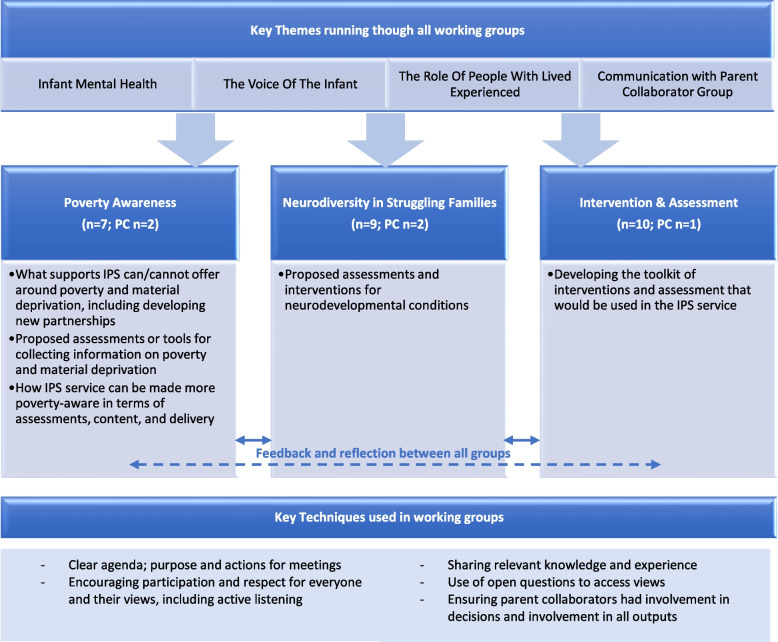
Working group composition and terms of reference

Further detail on our approach to co-production of IPS is provided in Pownall et al. (Pownall, Dalgarno, Graham, Crawford, Minnis: Lessons learned from co-producing a new intervention: the perceptions and experiences of professionals involved in the coproduction of a support service to improve the mental health of children aged 0–5 with a social worker, in preparation). and briefly outlined here. The IPS intervention was developed through working closely with service users and multi-agency professionals. Two groups of parent collaborators (PCs), one in Bromley and one in Glasgow, were formed with five parents with experience of child and family social work in each. These groups were led by our coinvestigator and lived experience lead (SG). Twenty-two multi-agency professionals including social workers, social work managers, members of the existing IFTs (comprised of clinical psychologists, child and adolescent psychiatrists, social work, and family liaison workers, all with special interests and direct experience of working with Infant Mental Health) were involved in the intervention development. A significant development that occurred during the early phases of the research was the formation of three working groups (Fig. 1) at which PCs and professionals worked together to refine IPS. These groups were invaluable in making sure the voice of lived experience remained at the core of our work and decision making. Groups met approximately fortnightly and to explore and discuss each aspect of the intervention in turn.

## Objectives {7}

The Feasibility RCT of IPS compared to local authority (LA) facilitated services-as-usual (SAU) commenced in April 2023 and will be completed in March 2024. The overall aim of the planned study is to investigate the feasibility of a definitive Randomised Controlled Trial (RCT) of IPS compared with services-as-usual (SAU), and of the planned study procedures for a future definitive RCT to evaluate the intervention’s clinical efficacy and cost-effectiveness. The main objective is to assess whether enough families can be recruited and retained such that a full-scale RCT is likely to be feasible. The secondary objectives are to assess the acceptability and feasibility of both the planned outcome measures for a definitive RCT and the IPS intervention to families and professionals. This information will be used to inform a decision on whether a full randomised controlled trial is warranted and achievable. Since this standalone feasibility RCT is designed to assess the feasibility and acceptability of the study design and data collection tools, it does not directly progress to a larger phase III RCT. However, predefined progression criteria are still necessary to guide the iterative process of the study. These criteria will be used to monitor and evaluate key aspects of the trial (e.g., recruitment, retention, data collection). The colour-coding system (green, amber, red) will be employed to monitor progress, with green indicating that outcomes are on track and requiring no changes, and amber/red signalling areas where adjustments or more significant modifications may be required. This iterative process aligns with the MRC framework for developing complex interventions [76], where feasibility findings guide necessary adaptations to the study design and methodology. A summary of the feasibility and process evaluation outcomes, along with the feasibility criteria and progression/adaptation criteria, is provided in Table 1.

**Table 1:** Main feasibility outcomes and progression criteria

Outcome	Elements	Measurement	Feasibility Criteria	Progression/Adaptation Criteria^a^
Recruitment, eligibility, and randomisation	Participant recruitment	• Number of families identified and assessed for eligibility • Numbers and percentage of families interested in participation of the total number identified	No criteria set	
		• Numbers and percentage of families interested, assessed for eligibility, meeting the inclusion criteria and included, of the total number identified	≥ 15 participants recruited per site, total 30 participants	**Amber**: If recruitment is below 30 families, adjust recruitment strategies or explore alternative pathways
		• Qualitative data on recruitment procedures and pathways (through interviews with the recruitment co-ordinator and professionals)	No criteria set	**Amber:** If significant barriers are identified, revise recruitment strategies or consider new outreach methods
	Participant eligibility	• Qualitative data on ambiguities regarding eligibility criteria, reasons for ineligibility and reasons for non-participation (through interviews with the recruitment co-ordinator and professionals)	No criteria set	**Amber**: Revise eligibility criteria or adjust strategies if significant ambiguities or issues are identified
	Randomisation	• Numbers and percentage successfully randomised at each trial site (target n15 at each site)	≥ 15 participants recruited per site, total 30 participants	**Green**: No adjustment needed if the target recruitment of 15 participants per site is met **Amber**: If randomisation sucess is lower than expected, revise recruitment or randomisation procedures
		• Qualitative data on participant understanding and acceptability of recruitment/ randomisation processes	No criteria set	**Amber**: Revise randomisation procedures or provide additional clarification if acceptability or understanding is low
Retention and attrition rates	Retention	• Number and percentage of families retained in the study for 3-6 months post randomisation	70% of participants retained at 3-6 months	**Green:** No adjustment needed if retention is at or above 70% **Amber:** If retention falls below 70%, explore reasons for attrition and adjust strategies (e.g., follow-up support) **Red**: If retention falls below 50%, review follow-up methods and consider additional participant engagement strategies
	Attrition	• Qualitative data on reasons for attrition (through interviews with the recruitment co-ordinator and professionals and quantitative data collected via online data management system)	No criteria set	**Amber:** If attrition is high, explore the reasons for attrition and revise follow-up methods or engagement strategies accordingly
Acceptability / engagement with IPS/ SAU	Acceptability of intervention components, barriers, and facilitators	• Qualitative data from interviews with participants and interviews and/or focus groups with professionals	No criteria set	**Amber**: Adjust the intervention components if feedback indicates low engagement or significant barriers to participation
Data Collection	Baseline and follow-up data collection procedures	• Quantitative data on questionnaire completion rates, errors, missing data from questionnaires • Length of time for participants to complete questionnaires / interviews	No criteria set	**Amber**: If completion rates or response rates are low, or if there are significant errors or difficulties in completing assessments, refine the data collection tools or extend the collection time
	Acceptability of research assessments and procedures Parent-reported outcome measure (PROM) and parent-reported experience measure (PREM)	• Qualitative data from interviews/focus groups with families and professionals • Qualitative interviews with families to assess acceptability and appropriateness for use in a definitive RCT		**Amber:** If qualitative feedback indicates unclear or inconsistent responses, or if there are significant challenges in engaging participants, consider refining the questions, methods, or extending the data collection time
Clinical efficacy and cost-effectiveness	Feasibility of collecting data on clinical efficacy and cost-effectiveness of IPS compared to SAU	• Data on resources used for delivering each arm of trial (to enable cost calculations) including group facilitator time and grade, training costs, staff / participant transport costs, time in intervention etc • Parent self-report questionnaire, capturing pre-post changes in service use, parental employment, leisure time and other personal costs (Demographic and Service Use Questionnaire)	No criteria set	**Amber**: If data collection on clinical efficacy or cost-effectiveness is challenging, revise or streamline data collection procedures, or assess alternative methods **Red**: If data cannot be collected, reconsider the methods
		• Qualitative data from interviews/focus groups with families and professionals		**Amber:** If qualitative feedback suggests difficulties in capturing meaningful data on cost-effectiveness or clinical outcomes (e.g., unclear responses or low engagement), consider revising the questions or methods, or exploring other ways to collect data **Red:** If data cannot be collected, reconsider the methods

^a^Green: No adjustment needed. The outcome is achieved or expected to be met; Amber: Minor adjustments are needed. The study will adapt to improve feasibility or engagement (e.g., modify recruitment strategies, refine intervention components); Red: Major adjustments needed. Significant issues require immediate intervention (e.g., revising strategies or core study components)

## Trial Design {8}

This multi-site single-blind randomised controlled trial (RCT) compares two services, IPS and SAU. The study will be a parallel, two-arm pilot RCT with a 1:1 allocation ratio across the two trial arms [1]. We will utilise a pre-post and 3/6-month follow-up design with embedded mixed-method process evaluation and exploratory economic analysis.

## Methods: Participants, Interventions and Outcomes

### Study Setting {9}

The study settings are social care services in the local authorities feeding into the two trial sites: Glasgow City Council (Scotland) and the London Borough of Bromley (England). Although each site is in the UK, the social services and legal contexts in each differ considerably. With regards to levels of deprivation, Bromley is a relatively small borough and relatively prosperous. Nonetheless, there are pockets of deprivation particularly in the North-East and North-West of the borough [77]. Glasgow City council is one of the largest local authorities in the UK. Although it does cover prosperous areas there are extensive areas of multiple deprivation [78].

### Eligibility Criteria {10}

#### Inclusion

At this early stage we have employed a broad eligibility criterion. Our target population is ‘children in need’ (or the Scottish equivalent); that is, “*a child who is unlikely to reach or maintain a satisfactory level of health or development, or their health or development will be significantly impaired without the provision of children’s social care services*” (p. 53) [79]. Eligible participants are families in the Glasgow or London trial sites where i) the infant(s) are aged 0–5 years (having additional children aged above 5 will not exclude the family from participating), ii) the family has an allocated social worker and plus a multi-agency support plan. The latter may include but is not limited to child and adolescent mental health services, paediatrics, or educational psychology, and where an infant and/or parent has a mental health problem or neurodevelopmental condition. Screening measures will be used to recruit families whose infants demonstrate some mental health and family relationships problems.

#### Exclusion

**Fig. 2:**
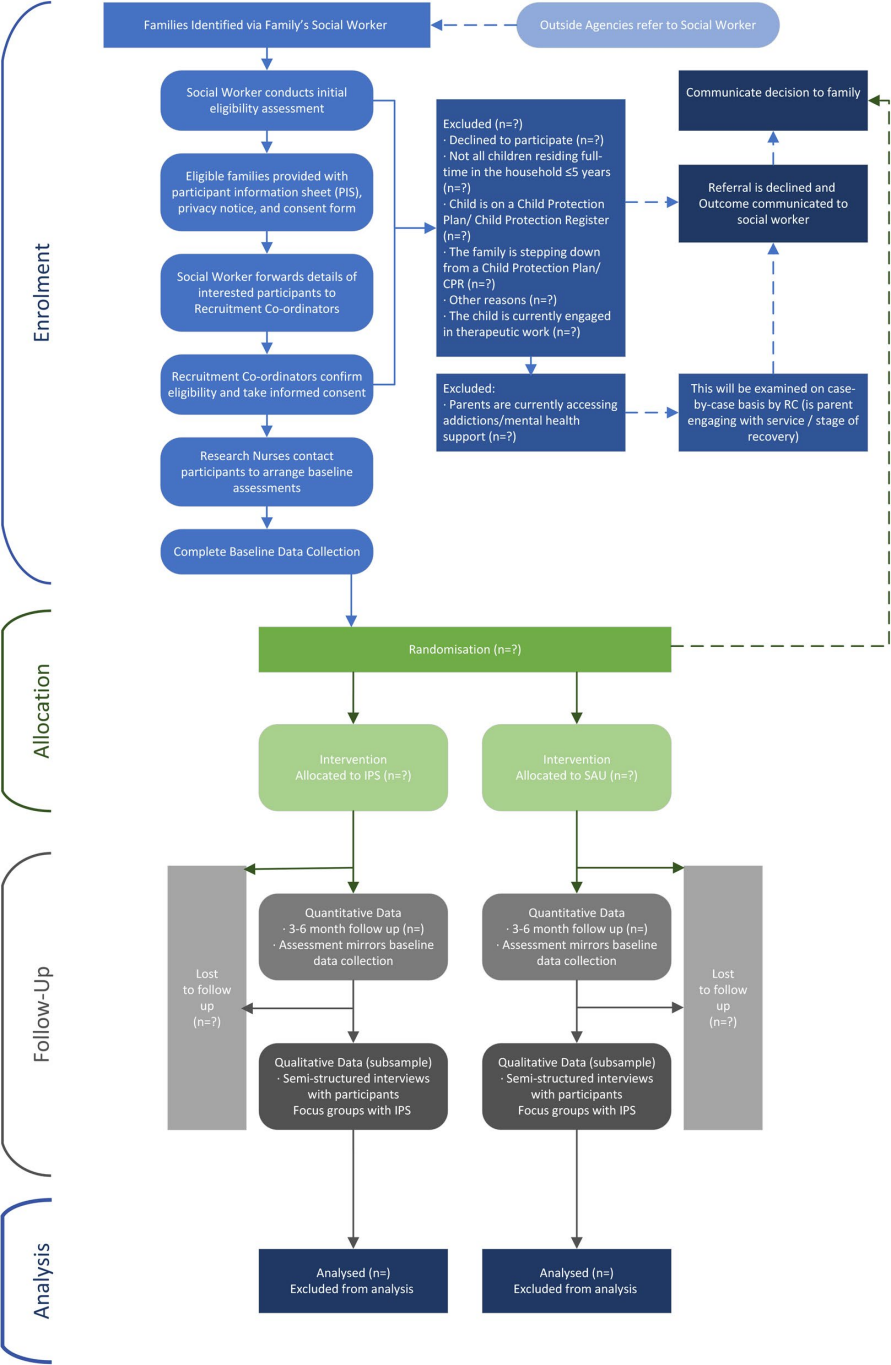
SPIRIT flow diagram of participants through the trial

IPS is an early intervention and the families who are likely to benefit from it are those whose problems are severe enough that IPS will offer tangible benefits, yet whose problems are not so entrenched that change is impossible. Therefore, potential participants will be excluded from the study if the infant has a Child Protection Plan (CPP) or is on the Child Protection Register (CPR), the family are in the process of ‘stepping down’ from a CPP or CPR, or if the infant is currently engaged in therapeutic work. For parent participants who are currently accessing addiction and/or mental health services, cases will be examined on an individual basis by the Recruitment Co-ordinator (RC; see Recruitment below). For instance, following a discussion with the recovery workers supporting the parent, the research team assessment regarding inclusion would be made based on the parent’s capacity to engage with therapeutic support and if this was viable alongside their existing recovery plan and treatment. The flow of participants through the study is shown in Fig. 2.

## Interventions

### Intervention Description {11a}: Infant and Family Support (IPS)

The experimental intervention is IPS, which aims to improve infant-parent relationships and reduce the risk of children needing to come into care for families who have a social worker. IPS is delivered by multi-disciplinary infant mental health teams operating to the NSPCC’s (National Society for the Prevention of Cruelty to Children) safeguarding and equality and diversity policies, with local authorities retaining ultimate safeguarding responsibility subject to English [79, 80] requirements. Within IPS, Family Engagement Workers (FEWs; discussed further below) have expertise-by-experience of having a child in need, other IPS practitioners have expertise-by-training and working in social work, psychology, or psychiatry, and some have both types of expertise.

IPS is derived from the New Orleans Intervention Model (NIM) [26] and has been extensively used by IFT teams [28]. As such, IPS professionals are already trained in the infant mental health assessment and interventions that will be used, although supplementary training was provided around poverty awareness and neurodevelopmental conditions. In addition, the co-production of IPS has led to adaptations to NIM, with the most significant changes being the addition of the Social Needs Questionnaire (SNQ) and ESSENCE-D [81] during the formulation phase. The SNQ assesses families’ personal, social, and financial needs, allowing the identification of supports required, as well as facilitating access to these supports as part of the intervention. Essence-D is a new tool designed to help practitioners to support families to develop a better understanding of either their child’s or their own neurodevelopmental profile. The information collected will be included in the formulation, and the intervention offered will be modified according to any neurodiversity needs identified.

Like the IFTs already operating in Glasgow and London, the IPS teams will use evidence-based relationship-focused interventions (up to 4 months) of the kind shown to reduce child abuse risk/increase parental sensitivity [82, 83]. This will ensure families receive a bespoke relationship-focused intervention that aims to be tailored to their unique needs. As the inclusion of FEWs to the IPS team is a new development (see below), there will be time allocated to induction and team building at the start. The family’s exit from IPS involves a “warm handover” to other local community services.

Using a poverty-aware and relationship-focused approach, the intervention will aim to improve infant mental health via improvements in the family’s material circumstances and infant-parent relationships – with the ultimate aim of the infant safely remaining within their birth family. Should child protection proceedings be required, learning about relationship quality and the degree of change achieved through intervention, will inform recommendations to the social care system.

#### Family Engagement Works and Family Engagement Leads

Our early co-production work highlighted the need for a Lived Experience workforce to help bridge the gap between IPS and the families that will use the service. As a result, the IPS teams will include two Family Engagement Workers (FEW) and a Family Engagement Lead. The Family Engagement Worker, informed by their own experiences of working with services, will work alongside families as they are randomised into IPS, discussing the intervention, seeking informed consent, arranging appointments (which could include preference for home visit or office appointments, transport/childcare needs, and identifying any learning, language, or cultural needs) and conducting some of the early assessments with families. In particular, the FEW will enhance the relationships between professionals (e.g., social workers and clinicians) and families through working with parents and caregivers, to promote choice and opportunities to enable families to feel connected and valued in their assessment and support plan. Family engagement is tailored to meet the local needs of families and support their connections with other helpful networks and services.

The Family Engagement Lead will ensure FEWs are fully supported to deliver safe, quality interventions with families within the wider team, and ensuring that decisions and practices are informed by lived experience at all levels. Their role will be to recognise and consciously work to remove barriers for the Lived Experience workforce, including practical constraints, attitudes/workplace culture, and bureaucratic processes that restrict effective and authentic Lived Experience work. Following the development phase establishing the need and readiness for this feasibility trial, this paper now outlines the protocol for the feasibility trial of IPS.

### Services as Usual {6b}

Families not randomised to IPS will receive services as usual (SAU), i.e., any assessment and/or support that social services and others normally implement when children have an allocated social worker. SAU includes regular contact with families by a social worker Case Manager, and may include signposting of families towards existing Health and/or third sector services depending on needs and availability of services. Families receiving IPS will continue to receive SAU in addition to IPS unless they were case closures to children services.

Services-as-usual (SAU) are often complex, involving health, social care and third sector services. In BeST^?^ [26] and our earlier work in this study [62] we created detailed maps of these local services, and have supported multi-agency partnerships between these services, in Glasgow and Bromley. Families’ journeys through SAU will be monitored, both qualitatively via continued systems mapping, and quantitatively via participant-level service use data also feeding into economic evaluation.

### Outcomes {12}

The primary outcome will be the success of recruitment and retention of 30 families.

The secondary outcomes are:

- Acceptability and feasibility of planned outcome measures for a definitive RCT (see below), including the newly developed parent-reported outcome measure (PROM) and parent-reported experience measures (PREM; see later)
- Acceptability of the IPS intervention to both families and professionals
- Perceived changes in the organisation, access, and quality of services, for children with a Social Worker
- Feasibility and acceptability of bespoke service use questionnaires, data collection forms and health related quality of life (HRQoL) measures to inform the future cost-effectiveness of IPS versus SAU.

#### Primary Outcome Measure

Our primary outcome measures will capture the following elements (Table 1):

- Can we identify and randomise 30 eligible families across Glasgow and Bromley? *This will capture the proportion of eligible families that consented to the trial, as well as qualitative data on recruitment procedures and pathways.*
- Can a sufficient proportion of these 30 families be retained in the study for 3 and/or 6 months post randomisation? *Quantitative data will include proportions of families retained in the trial, and qualitative data will focus on reasons for attrition.*

We will also collect data on any ambiguities regarding eligibility criteria, reasons for ineligibility and non-participation, to inform the design and conduct of a definitive trial.

#### Secondary Outcome Measures

Our secondary outcomes will focus on the acceptability and feasibility of both the proposed evaluation methodology and the intervention itself. This will predominantly be examined through a detailed process evaluation (see below), although quantitative data regarding assessment completion rates, errors, and missing data, as well as the differential impact of face to face and remote assessments will also support our analysis.

#### Putative Outcome Measures for Definitive Trial

**Fig. 3:**
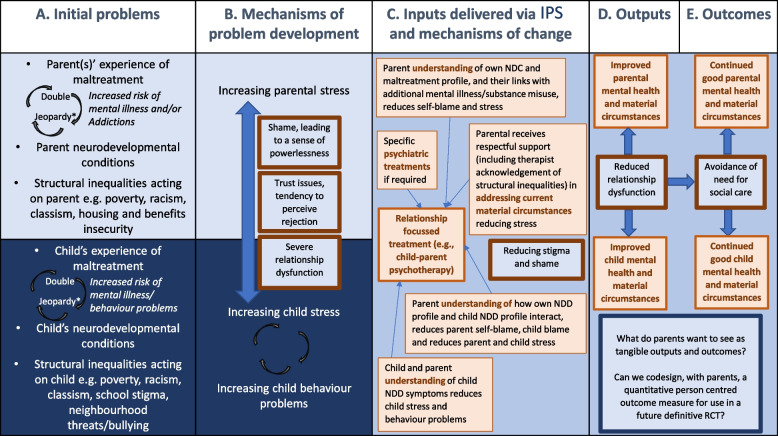
The logic model, IPS, *Gajwani R and Minnis [84]

The following outcome measures have been selected with the aim of addressing the key components of our IPS logic model (Fig. 3) and will be administered to all families at pre-randomisation and 3-to-6 months follow up to determine the feasibility and acceptability of their inclusion for a definitive trial. These measures have been selected through our review of the literature and some are being used in ongoing RCTs in similar populations [26] (Table 2).

**Table 2:** Study Procedures

**Activity / Assessment**	Staff Member	Initial Referral	Prestudy screening/consent	Prestudy Baseline assessment	Randomisation	Follow-up 3/6 months postbaseline
Eligibility Assessment	SW	✔				
Participant Information Sheet	RC		✔			
Consent^1^	RC		✔			
Privacy Notice	RC		✔			
Randomisation	TM				✔	
***Infant-based measures***						
b-CAPI * To be completed for all children*	RN			✔		✔
SDQ * To be completed for children aged 2 and over, according to age of child:* * ◦ 2-4y, 4-17y*	RN			✔		✔
ITSEA * To be completed for children aged 2 and under*	RN			✔		✔
PEDs-QL * To be completed for all children, according to age of child:* * ◦ 1–12 m, 13–24 m, 2-4y, 5-7y*	RN			✔		✔
***Parent-based measures***				✔		✔
Demographic and Service Use Questionnaire	RN			✔		✔
GHQ-12	RN			✔		✔
ICECAP-A	RN			✔		✔
PREM	RA/FEW			✔		✔
PROM	RA/FEW			✔		✔
Parent Qualitative interview	RA			✔		✔
***Professional-based measures***						
Professional Focus group Parent Collaborator Focus group / interview	RA RA					✔ (+ as required) ✔ (+ as required)

Key: ^1^Consent will take place a minimum of 24 h after information has been presented to families

*SW* Social worker, *RC* recruitment co-ordinator, *TM* trial manager, *RN* research nurse, *RA* research associate, *FEW* family engagement worker, *b-CAP* Brief Child Abuse Potential Inventory, b-CAP, *SDQ* Strengths and Difficulties Questionnaire, *ITSEA* Infant–Toddler Social & Emotional Assessment, *PEDs-QL* Paediatric Quality of Life Inventory, *GHQ-12* General Health Questionnaire, *ICECAP-A* ICEpop CAPability measure for Adults, *PROM* parent-reported outcome measure, *PREM* parent-reported experience measure

##### Infant Mental Health

In a definitive trial our primary outcome will be infant mental health and our planned principal outcome measure will be the Brief Child Abuse Potential Inventory (b-CAP; [85]). This is a parent-report instrument that measures risk of child maltreatment. Secondary outcomes will be the Paediatric Quality of Life Inventory (PedsQL [86]), which assesses health-related quality of life and, depending upon the child’s age, either the Strengths and Difficulties Questionnaire (SDQ [87]) or the Infant–Toddler Socio-emotional Assessment (ITSEA [88]) to characterise children's Social–Emotional Development (Table 2).

##### Parental Mental Health

Parent mental health will be captured though the General Health Questionnaire (GHQ-12 [89]) which is a well validated measure of nonspecific psychological distress. In addition, the ICEpop CAPability measure for Adults (ICECAP-A, [90]) will be assessed and can be used to calculate parent utilities in the economic analysis. The ICECAP captures attributes of wellbeing which are important to adults so has a broader definition of QoL than the GHQ which focusses on health.

##### Parent-Reported Outcome Measure and Parent-Reported Experience Measure

In addition to the validated outcome measures described above, an important output of our earlier co-production and qualitative process evaluation work was the development of a parent-reported outcome measure (PROM) and parent-reported experience measure (PREM) to capture outcomes and experiences that are salient to our families when engaging with social care. These measures will be piloted with families in the current study and completed with the researcher and lived experience lead.

PROMs have been successfully used in healthcare to inform shared decision-making and to tailor care to individual needs, and are increasingly used in randomised controlled trials to examine whether an intervention is effective and cost-effective from the perspective of service-users [91]. PREMS are used to examine patients’ experiences of healthcare and examine factors such as empathy and respect [92]. Although the role of PROMs and PREMs in health care is increasingly being recognised, to our knowledge this will be the first time PROMS and PREMS have been developed for a social care context. In line with the theoretical basis for this study that highlights how stigma, shame, and structural inequalities are key problem drivers, and that respect, reduction in shame, and development of trust are crucial in allowing relationship-focused interventions to be effective, we believe that development of these new tools will be essential. The data from the PROM and PREM will also feed into the economic evaluation (see below).

#### Data Capture on Resource Use for Service/Intervention Delivery for IPS/SAU

In a future trial we will undertake a full economic evaluation and decision analytic model, exploring the potential cost-effectiveness of IPS in comparison to SAU. Currently, we will test our planned health economic methods for use in definitive evaluations, including the feasibility of collecting data on (i) intervention delivery in IPS and SAU, (ii) self-reported service use, and (ii) quality-of-life measures. To examine resource use associated with IPS, as well other service impacts, we will generate data from NSPCC system routine data (e.g. data on number of meetings, attendees, duration, training, and other costs) to calculate a unit and total costs for future trials. In a future definitive study, we would explore linkage with routine NHS data to allow validation of participant-report data to allow a full economic model to be constructed. In order to get resource use information for the SAU arm, a data collection form was adapted from a previously developed form for the BeST^?^ Trial [26]. Measurement of quality of life is an important outcome for the economic component of this study and will enable exploration of potential short-term changes in quality of life for children between baseline and 3-6 months, and between the trial arms. The ICECAP-A will also be used to inform the economic evaluation, deriving utility values for the parents.

##### Demographics and Service use Questionnaire

Based on prior experience from the BeST^?^ Trial [26], we anticipate potential challenges in obtaining resource use data from services as usual. Therefore, we plan to additionally capture parent reported use of services in each arm as an alternative data source. This will document use of health, education, and social care service use both within IPS and SAU, as well as the parent(s) demographics and circumstances such as employment, benefits, and housing. This will be collected at baseline and follow up (3-6 months).

### Process Evaluation

**Fig. 4:**
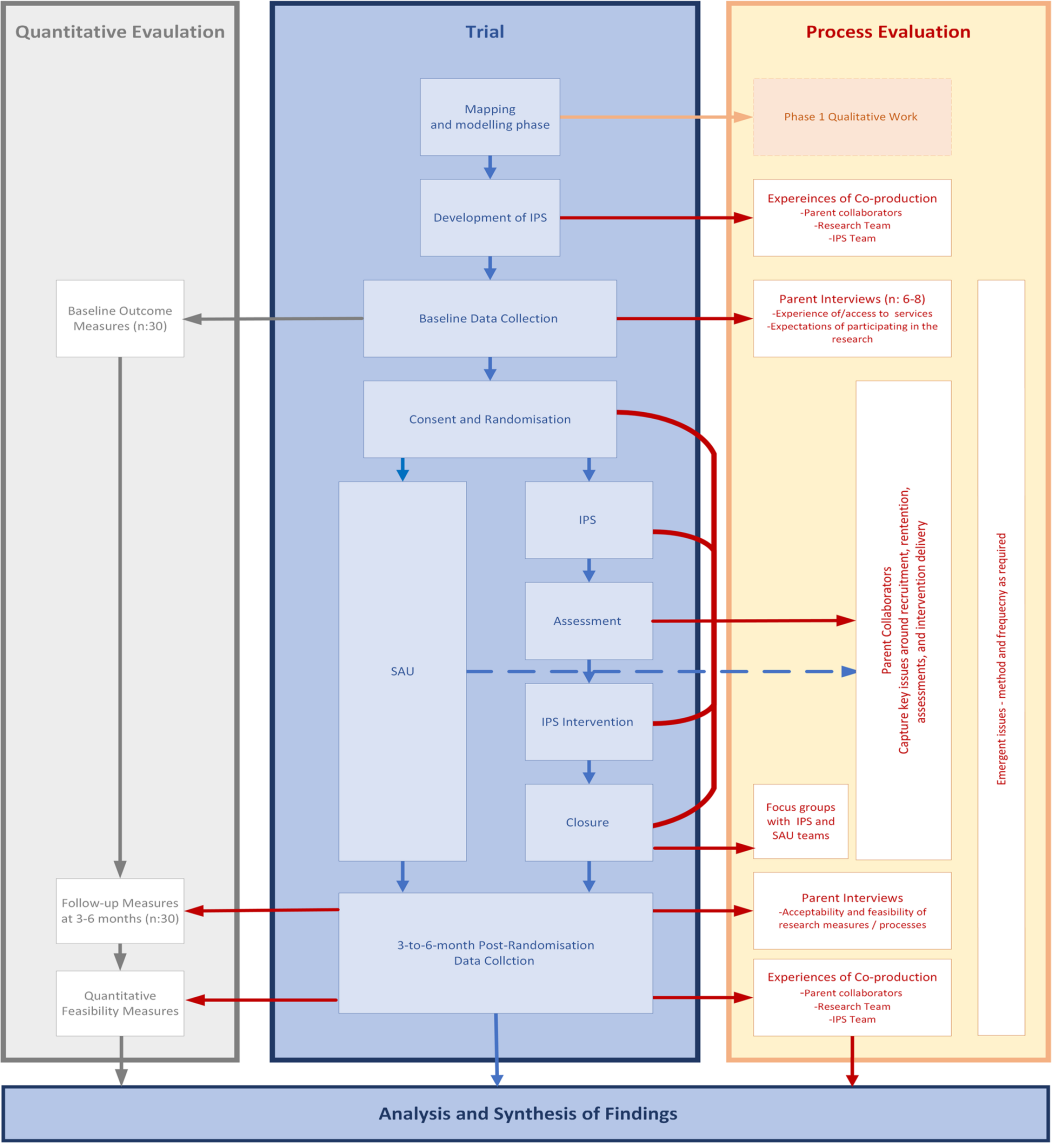
Overview of the partnership for change study

A detailed process evaluation will run parallel to the trial, following the Medical Research (MRC) process evaluation framework for Complex Interventions which emphasises the importance of considering interactions between implementation, mechanisms, and context [93]. This will enable a rich understanding of participating families and key stakeholders’ acceptability and feasibility of the proposed evaluation measures, as well as capturing issues around the implementation of IPS as they unfold within the current context. Estimates of treatment effectiveness should not be the aim of feasibility trials [94], however, through a carefully conducted process evaluation we can track the ways in which IPS evolves and impacts as a service, capturing and exploring issues as they arise throughout the trial and feeding into service development. The process evaluation will be conducted at regular timepoints, capturing the early stages of IPS delivery and how it evolves as a new service from multi-perspectives (Fig. 4).

#### Parents

Parents taking part in the study (IPS or SAU) will be invited to participate in up to two semi-structured interviews. The first interview will take place soon after baseline and will explore a sample (n6-8) of parent’s experience of, and access to mental health services and expectations of participating in the research. At follow up of 3-to-6 months, all parents will then be asked to take part in a second interview, centring around their engagement with the research process, including their experience and understanding of the randomisation and consent process, suitability of the assessments (including the PREM and PROMs), and practical aspects of participating in the research. Individual one-to-one interviews are the preferred method of data collection given the potential sensitivity of topics raised and the need for flexibility.

#### Parent Collaborators

The co-production values of equality, diversity, access, and reciprocity [65] run throughout all phases of the trial, ensuring that individuals who have been previously silenced by structural inequalities are able to have a voice and an equal role in shaping the IPS teams. Parent collaborators will continue to take an active role during the intervention roll out phase, providing advice and guidance on the conduct of the trial, such as resolving difficulties around recruitment and retention and engagement with IPS or the research process. Learning will be captured through meeting minutes and strategically placed focus groups or interviews, for example following the assessment phase of IPS to capture challenges, modifications, and improvements as the service evolves (Fig. 4).

#### IPS and Services as Usual Practitioners

Focus groups will be conducted with each of the teams delivering IPS and SAU across both sites, including Family Engagement Workers and Family Liaison Workers (the latter in Glasgow only) focusing on the perceived changes in the organisation, access, and quality of services. Practitioners will be encouraged to explore referral pathways into IPS or other services within SAU, the perceived acceptability of intervention components, including any drivers and barriers to successful implementation (from their own or the families’ perspective), how it fits within the local context, highlighting potential differences across settings, as well as perceptions around the research process itself. Focus groups are particularly useful for professional or other groups used to sharing experiences, allowing the group dynamic to facilitate the safe emergence of different perspectives [95].

#### Emergent Issues

Through an iterative process of data collection and analysis, any emergent issues or unforeseen difficulties that arise from the process evaluation will be explored further as the trial progresses. These findings may highlight the need to interview other professional groups or stakeholders who had not been formerly considered. Previous work has highlighted how qualitative approaches can be powerful in developing new and strengthening existing relationships across groups and agencies [96]. Utilising an iterative approach will be fundamental to identifying potential sites for a definitive RCT. Findings will be shared across parent collaborators and professional stakeholder groups to identify whether barriers and facilitators to services can be refined and developed into codesign responses.

### Participant Timeline {13}

Study procedures are summarised in Table 2.

### Sample Size {14}

We plan to recruit 30 families from Glasgow or London, with 15 families randomly allocated to IPS and 15 to SAU. Since the primary aim of this study is to assess recruitment rates, retention rates, and the acceptability of proposed measures, a formal power calculation is not appropriate. Our sample size was chosen to ensure a meaningful assessment of feasibility. If the true retention rate is 70%, the 90% confidence interval for this estimate will be approximately ± 15%, which we consider sufficiently precise for a feasibility study [97]. This level of precision allows us to make an informed decision about the feasibility of a larger trial. Additionally, as Hertzog (2008) [98] notes, the sample size for pilot studies should balance precision with logistical constraints, which we have considered in our planning. Furthermore, if the true retention rate is 70%, there is an > 80% probability that the observed retention rate will exceed 60%. If the observed retention rate falls below 60%, this would suggest that a future full-scale study is unlikely to be feasible without modifications. Even if feasibility is demonstrated, any future trial will include an internal pilot phase with close monitoring to ensure robust outcome data can be collected at scale.

### Recruitment {15} and Consent {26}

Pathways into the trial have been defined collaboratively with Parent Collaborators, local authority partners in Glasgow and Bromley and the IPS teams (Fig. 2). This collaborative approach ensures that recruitment into the sample is representative of our target population, in terms of social class, ethnicity, gender, sexuality and disability. Our recruitment and retention strategy builds upon a highly successful approach used in our ongoing BeST^?^ trial, employing experienced social work-trained Recruitment Coordinators [99]. The trial recruitment coordinators (RCs) will work with organisations to identify the target population, facilitate referrals to the study, and screen for eligibility. Parent collaborators will also be instrumental in supporting all participants throughout the research process, including any who withdraw.

Potential participants may be identified by social workers (and other support services via a social worker) who will conduct the initial eligibility assessment and provide families with the study participant information leaflet explaining the study, its intent, and what participation would entail. With the family’s consent, their details will be passed onto the RCs who will talk to the interested families to discuss the study further, answer any questions and, if proceeding, go through the informed written consent process. Consent to access routinely collected health and social work data about the child and parent will also be sought, to test the feasibility of collecting data on service/intervention delivery for each arm.

The research nurse (see below) will contact Consenting families and invite them for baseline research assessments (Table 2). Once this is complete, they will be randomised to IPS or services-as-usual. Families will be given the option of assessments taking place remotely or face-to-face. The flow of participants through the study is shown in Fig. 2. An online system will collect eligibility screening data for referrals, providing data on eligibility rates, any ambiguities regarding eligibility criteria, reasons for ineligibility, and non-participation. This will also alert the research team to situations when referral and/or recruitment rates fall below expectations to allow remedial actions if required. After randomisation, the RC will pass the referral information to the IPS Team Managers, and the IPS Family Engagement Worker will contact the family to commence assessments. In both arms of the trial the RC will inform the family and SW of randomisation outcome (see below).

For the process evaluation, all parents taking part in the study will be invited to participate in a semi-structured interview. These will be conducted in a separate session at a mutually convenient time and place, by virtual or face to face methods. These interviews will be optional, and a member of the research team will take written informed consent separately. Participants will receive a voucher for taking part in research interviews. In relation to parent collaborators and IPS and services as usual practitioners, informed consent will be taken prior to conducting any interviews or focus groups. It will be emphasised that participation is voluntary, and data will be anonymous. Only the researcher conducting the process evaluation will know which members of the teams will and will not participate.

### Assignment of Interventions: Allocation {16} and Blinding {17}

After informed consent, verification of inclusion/exclusion criteria, and completion of baseline measures, participants are stratified by site (Bromley or Glasgow) then individually randomized within site to either IPS or SAU (1:1) via computer-generated randomization through www.graphpad.com. Once a family has completed their baseline assessments, the recruitment coordinator (RC) will notify the trial manager, who will then confirm the assigned trial arm via email. During the feasibility stage, the randomisation sequence will have been predetermined and securely recorded, accessible only to the trial manager. This process will ensure allocation concealment, preventing prediction or influence on group assignments. The RC will then inform the family and referrer of the randomisation outcome via telephone, text, or email. The study will follow a single-blind design: participants and providers will be aware of the treatment allocation after baseline assessments, whereas researchers conducting baseline and follow-up assessments will remain blinded. To maintain blinding integrity, researchers will follow a scripted protocol when conducting assessments, as in the BeST^?^ Trial. Additionally, at the end of the trial, researchers will state which intervention they believe parents received to assess the effectiveness of blinding.

### Data collection and Management {18–19}

Research nurses who will be conducting the baseline assessment will be trained/ experienced in administering the study measures. All instruments will be uploaded into the Qualtrics platform for online surveys (www.qualtrics.com). For the measures requiring rating, more extensive training will be offered until raters achieve sufficient reliability.

#### Confidentiality {27}

All data collected will be kept securely and separate from any individual participant identifiers. Participants will be assigned a unique ID number to link their data throughout the trial.

### Statistical Methods {20A}

#### Quantitative Analyses

Descriptive statistics will be used to report the primary outcome of participant retention and recruitment. Retention will be presented as a simple percentage of participants randomised who remain in the study at 3/6-month end point. Recruitment will also be presented as a number, however, to capture movement through the research process, numbers and percentages will also be presented to summarise potential participants identified, invited, assessed for eligibility, eligible, and included and randomised into the study (Table 1). Data will also be presented for reasons for ineligibility, ambiguities regarding eligibility criteria, reasons for non-participation, and mode of assessment administration (face to face or remote). Reasons for exiting the study will be collected throughout the trial.

To supplement the process data of acceptability and feasibility of the outcome measures, the mean and standard deviations for the length of time taken for participants to complete baseline and follow-up assessments and errors and missing data will be captured. Comparisons will be made between face to face and remote assessments. For the putative outcome measures, the mean scores and standard deviations will be presented.

#### Qualitative Analyses

Data are audio-recorded, transcribed, anonymised, and analysed using the qualitative data management software NVivo 12 (QSR International, Warrington, UK). Data are analysed thematically following Braun and Clarke’s steps which include independent reading of transcripts to develop a coding frame, review and naming of codes, and development and reporting of key themes [100]. This is an iterative process and is continually reviewed and agreed upon as the research progresses to ensure that it continues to meet our research needs.

## Discussion

The English Care Review Case for Change [32] and The Scottish Care Review (The Promise) [33] highlight early intervention as a cornerstone for achieving better outcomes for children and families, yet there is a paucity of research on effective approaches to preventing child maltreatment. The aim of the Partnership for Change project is twofold. First, to co-produce a new service, Infant Parent Support (IPS) with parents who themselves have had children involved with social work. Secondly, and the focus of the current paper, to conduct a feasibility RCT of this service for infants who have social work involvement, but do not yet have a child protection measures in place, and their parents. An early intervention like IPS, that uses a relationship-focused approach to infant and parent mental health, neurodevelopmental and money/housing problems, while developing and strengthening multi-agency partnerships, has never been developed before. The results of this study will ascertain whether we can conduct a definitive RCT evaluating IPS intervention against services-as-usual to improve the mental health of children.

Findings from the mapping and modelling phase of this study have showed excellent promise and positivity around the trial, with local authorities, stakeholders and parents wanting to engage in its delivery. We have built a detailed picture of how child and adult health and social services work in the contrasting legal and social care contexts of Glasgow and London, demonstrating that care pathways are adequate to ensure safe delivery of IPS (i.e., sufficient multi-agency communication and planning to ensure child safety [62]. This work has also highlighted how societal, systemic, service, and personal barriers can impact upon families’ journeys into and out of services which have informed the trial.

Given the uniqueness of the intervention and the target population, assessing the acceptability and feasibility of the programme and evaluation methodology is fundamental. Progression criteria to a full RCT is based on adequate recruitment and retention into the study. Nonetheless, individuals using social care services may encounter numerous barriers to participating in research, services, and interventions, for example concerns over confidentiality, issues of unequal power, and stigma [101]. The current study will allow information to be gathered to determine the most optimum recruitment and retention strategies to help understand and overcome these challenges, allowing design optimisation in future RCTs.

The embedded process evaluation will not only assess the acceptability of each measure to participants but will also examine the assessments’ ability to capture important outcomes of IPS. Central to this work is developing two new measures, the PREM and PROM, which will be used in a definitive trial to examine whether IPS is effective and cost-effective from the perspective of service-users. To our knowledge, this will be the first time PROMS and PREMS have been developed for a social care context.

We will explore qualitatively the perspectives of those implementing, delivering, and receiving the interventions as part of investigating the feasibility of implementing the model of intervention. These data will inform the optimisation of intervention delivery and understanding the barriers to and facilitators of implementation of IPS. As a new service, it is essential to capture how IPS will work within complex social work systems and multi-agency contexts. The findings of the process evaluation will also allow refinement of the intervention theory of change; that is the theory about how and why our intervention is expected work [102].

Although we will not explore intervention fidelity in this phase, our process evaluation will inform what data are needed to ascertain and optimise adherence to IPS in future trials. In addition, we will explore the potential of collecting resource use data, and quality of life data which will allow us to assess the feasibility, and set up systems to develop a full health economics analysis in a definitive RCT.

## Conclusions

Interventions aimed at preventing child maltreatment are only modestly effective [23, 103]. The current study attempts to address some of the limitations of previous interventions by developing a service that supports families when they first display signs of struggling, whilst also recognising the importance of supporting parent and child NDCs and incorporating a holistic poverty-aware approach [51, 52]. Above all, this new service will be co-produced by parent experts-by-experience, researchers, local stakeholders (e.g., social work managers) and infant mental health practitioners to maximise the potential for it to be accessible, timely, and relevant. Although the involvement of people using services in designing and delivering support can benefit individuals, professionals, services, as well as practice and policy [68], there remains a paucity of studies examining the impact of co-produced approaches on health outcomes [104, 105]. This study offers a first step in helping to address that gap.

Child maltreatment and subsequent care placement is profoundly costly for the children involved, for their families, and society [106–108]. Providing early interventions and support to struggling families could potentially lead to a long-lasting benefit for children and their families, as well as a reduction in costs to society [108]. Intervening before families get to crisis point is an ongoing priority, and is right for the child, family, and society [109].

### Dissemination Plans {31a}

A publication plan is reviewed at each Trial Steering Committee meeting and all submitted publications are logged with the funder. In addition, a dissemination plan will be co-produced with parent collaborators to ensure results are made available and accessible.

## Data Availability

Not applicable.

## Abbreviations

ADHD: Attention Deficit and Hyperactivity Disorder
ASD: Autism Spectrum Disorder
b-CAP: Brief Child Abuse Potential Inventory
BeST^?^: Best Services Trial
CPP: Child Protection Plan
CPR: Child Protection Register
FEW: Family Engagement Workers
GHQ-12: The General Health Questionnaire
HRQoL: Health Related Quality of Life
ICECAP-A: ICEpop CAPability measure for Adults
ID: Intellectual Disability
IFT: Infant and Family Team
IPS: Infant Parent Support
ITSEA: The Infant–Toddler Socio-Emotional Assessment
MRC: Medical Research Council
NDC: Neurodevelopmental Condition
NIHR: National Institute for Health Research
NIM: New Orleans Intervention Model
NSPCC: National Society for the Prevention of Cruelty to Children
PREM: Parent-Reported Experience Measures
PROM: Parent-Reported Outcome Measure
PC: Parent Collaborator
PedsQL: Paediatric Quality of Life
RC: Recruitment Co-ordinator
RCT: Randomised Controlled Trial
SAU: Services as Usual
SCIE: Social Care Institute for Excellence
SDQ: Strengths and Difficulties Questionnaire
SNQ: Social Needs Questionnaire
UK: United Kingdom
US: United States
